# Irritable bowel syndrome among Norwegian nurses – associations with insomnia, excessive sleepiness, shift work disorder and shift work schedule

**DOI:** 10.1186/s12912-026-04402-8

**Published:** 2026-02-12

**Authors:** Siri Waage, Ståle Pallesen, Bjørn Bjorvatn

**Affiliations:** 1 Norwegian Competence Center for Sleep Disorders, Haukeland University Hospital, Jonas Lies vei 65, Bergen, 5021 Norway; 2 Department of Global Public Health and Primary Care, University of Bergen, Bergen, Norway; 3 Department of Psychosocial Science, University of Bergen, Bergen, Norway

**Keywords:** Irritable bowel syndrome, Sleep, Sleepiness, Shift work

## Abstract

**Background:**

The present study aims to investigate the prevalence of irritable bowel syndrome (IBS) among nurses, and its associations with sleep- and sleepiness problems and shift work, as both shift work and poor sleep have been linked to high prevalence of gastrointestinal symptoms and IBS.

**Methods:**

The data stem from 1335 Norwegian experienced nurses participating in the cohort “SUrvey of Shift work, Sleep and Health” (SUSSH). Results are based on cross-sectional data collected in the 10th wave (2018) of SUSSH. The nurses responded to a questionnaire that assessed symptoms of IBS according to the Rome IV diagnostic criteria, symptoms of insomnia measured with the Bergen Insomnia Scale (BIS), excessive sleepiness measured with the Epworth Sleepiness Scale (ESS), shift work disorder (SWD) and shift work schedule such as day vs. night work, number of nights and number of quick returns (less than 11 h between two shifts) worked the last year. Chi-square tests were used to compare categorical variables and crude and adjusted (for sex and age) logistic regression analyses were conducted with IBS as dependent variable.

**Results:**

A total of 6.3% of the nurses reported IBS, 30.9% insomnia, 25.6% excessive sleepiness and 33.6% shift work disorder, respectively. In total 59.8% were working daytime schedules, while 40.2% had a work schedule that included night work. IBS was more prevalent among nurses with insomnia (no/yes) (4.8% vs. 9.7%, *p*<.001), excessive sleepiness (no/yes) (4.9% vs. 10.7%, *p*<.001), and shift work disorder (no/yes) (5.0% vs. 9.1%, *p*=.004), but not among nurses working night shifts (day work/night work) (5.9% vs. 7.2%, *p*=.351). The adjusted logistic regression analyses showed that insomnia (aOR 2.14, 95% CI 1.36–3.67), excessive sleepiness (aOR 2.36, 95% CI 1.48–3.75) and shift work disorder (aOR 1.95, 95% CI 1.24–3.06) remained significantly associated with IBS.

**Conclusions:**

In this population of experienced nurses, the prevalence of IBS was quite low. However, IBS was more prevalent among nurses having sleep problems like insomnia, excessive sleepiness and shift work disorder, whereas work schedule was unrelated to IBS.

**Clinical trial number:**

Not applicable.

## Background

Shift work is described as work schedules where workers succeed one another temporally at the workplace in order to maintain continuous operation [1]. With need for 24/7 services in the health care sector, nurses are heavily involved in shift work such as night work and quick returns (QRs) defined as less than 11 h between two shifts [2]. Shift work disrupts biological rhythms and is associated with a wide range of negative health outcomes including both sleep and gastrointestinal problems [3]. The most commonly reported sleep problems related to shift work comprise problems maintaining sleep, reduced sleep duration, and excessive sleepiness during work [4]. Insomnia is prevalent among nurses, with estimates as high as nearly 39% [5]. Among Norwegian nurses, prevalence of insomnia has been reported to be as high as 52% and 55% [6, 7]. Also, work schedules that disrupt the circadian rhythms are associated with shift work disorder (SWD), a circadian rhythm sleep wake disorder characterized by symptoms of insomnia and/or excessive sleepiness temporally associated with a recurring work schedule that overlaps the usual time for sleep accompanied by a reduction of total sleep time [8–10]. Additionally, shift work is associated with gastrointestinal complaints like heartburn, abdominal pain, stomach ulcers, constipation and diarrhoea [11]. Such symptoms also overlap with those reported by patients with functional bowel disorders [12].

Irritable bowel syndrome (IBS) is a common gastrointestinal (GI) disorder in the general population. A meta-analysis including 80 studies based on both Rome I, Rome II, Rome III, Manning criteria and questionnaire-defined criteria reported a global prevalence of 11.2% (95% confidence interval: 9.8%−12.8%) [13]. It is defined by recurrent abdominal pain related to defecation or a change in the frequency or appearance of stool. The pathogenesis of IBS is not clear, but the prevalence varies across geographical locations, and is reported higher among women compared to men [14]. Since 1989, a sequence of consensus-based Rome criteria for IBS has been published, with the last update and fourth revision in 2016 [14].

Furthermore, IBS is linked to poor sleep. Several studies have reported an association between sleep disturbances and functional gastrointestinal disorders [15, 16]. A systematic review with meta-analysis from 2018 including 36 studies reported a prevalence of a sleep disorder in IBS of 37.6%, varying from 7.1% to 73.9%, where the majority of the studies (21 of 36) used the Rome III criteria [17]. In one study among Chinese nurses with poor sleep, IBS was reported in 35.1% when defined according to the Rome III criteria [15]. In addition, circadian disruption due to shift work is likely to predispose shift workers to an increased risk of GI disorders like IBS [11, 18]. The prevalence of IBS defined by Rome III criteria in rotating shift workers is reported to be nearly twice the prevalence of day workers (32.7% vs. 16.7%) in a Korean study [19]. Moreover, in US nurses, rotating shift work is associated with a significantly higher prevalence of IBS (Rome III) compared to day work, with prevalence rates of 48.0% vs. 31.0%, respectively [12]. The risk of IBS contingent on shift work has also been attested to by a recent large population-based cohort study [20]. However, one American study found no associations with overnight call work and IBS [21]. Still, a meta-analysis including eight studies concluded that shift work probably represents a risk factor for IBS, but that the strength of the evidence is limited and this calls for further studies on this topic [22].

Circadian disruption and poor sleep may impair IBS by disturbing the gut–brain axis signaling [23]. Consequently, this could lead to altered gastrointestinal motility, increased intestinal permeability and triggering stress-related elevated cortisol levels. These factors also affect autonomic balance, melatonin levels, and gut microbiota. Based on the assumption that both sleep problems and shift work are associated with IBS, the aim of the present study was to investigate the prevalence of IBS among a large group of experienced shift working nurses, as well as to explore IBS and its associations with shift work, sleep- and sleepiness problems. We hypothesized that nurses with shift work schedules leading to disrupted circadian rhythms, in particular night work and quick returns would report more IBS compared to nurses working day shifts. We also hypothesized that nurses with sleep or sleepiness problems would report a higher frequency of IBS compared to nurses not having sleep or sleepiness problems.

## Methods

### Procedure and participants

This paper is based on cross-sectional data from the cohort “**SU**rvey of **S**hift work, **S**leep and **H**ealth (SUSSH)” conducted among Norwegian nurses. The first data collection took place during winter 2008/2009 (wave 1), when a sample of 5400 nurses was randomly selected from the Norwegian Nurses Organisation’s (NNO) membership roll and asked to participate in the survey. A total of 2059 nurses responded to the questionnaire at the first wave (2008/2009), yielding a response rate of 38.1%. To increase the sample size, an additional sample of 905 newly educated nurses (response rate = 33.0%) was recruited in 2009, consequently the total sample in the first wave of the cohort included 2964 nurses. Except for nurses who have withdrawn from the study, died, or have unknown addresses, all nurses who responded to the first wave have since been invited to partake in annual follow-up survey waves. For each wave, the questionnaires have comprised a mix of both recurrent and new questions. Questions about shift work schedule, sleep and sleepiness, circadian rhythms and different health related factors have been included regularly. The questionnaires have been sent by postal mail with pre-paid envelopes for returning the completed questionnaire with up to two reminders to nurses not responding. For each wave, nurses completing and returning the questionnaire participated in a lottery, where 25 individuals won a gift card with a value of 500 NOK (~ 50 USD). The response rates in the follow-up questionnaires have been high, between 61% and 81%. The present study reports findings from wave 10 (2018), in which questions about irritable bowel syndrome were included for the first time. A total of 1698 nurses responded to the questionnaire, yielding a response rate of 61.2%. Only nurses who reported that they were working active as nurses at the time of the data collection were included in the analyses, resulting in an analytic sample of 1335 nurses.

## Instruments

### Demographics and working time variables

Except for data on sex and age that were collected in the baseline wave in 2008/2009, the 2018-questionnaire (wave 10) included questions related to the work schedule (response alternatives; day only, evening only, two-shift rotation including day and evening shifts, night only, three-shift rotation including day, evening, and night shifts, and other schedules with night work), numbers of nights and number of quick returns (QRs, less than 11 h between two shifts) worked the last year. Day only, evening only, and two-shift rotation were defined as day work, whereas three-shift rotation, night only and other schedules with night work were defined as night work in one dichotomous variable. For the analyses, the sample were divided into three groups related to numbers of nights worked the last year (0, 1–20, and > 20), and number of QRs worked the last year (0, 1–20, and > 20).

### Irritable bowel syndrome (IBS)

A set of previous developed questions [24] designed to identify IBS according to the Rome IV diagnostic criteria [25] were used to assess IBS. The questions were phrased; (I) Do you have abdominal pain at least one day a week (response alternatives; no, yes, do not know), (II) For how long have you had this pain (response alternatives; less than 1 month, 1–3 months, 3–6 months, over 6 months), and (III) If you have abdominal pain, do you have at least one of three episodes of this pain in connection with: (a) having bowel movements, or just before or just after a bowel movement? (response alternatives; no, yes, do not know), (b) bowel movements being looser or harder than usual? (response alternatives; no, yes, do not know), and (c) having bowel movements more frequently or less frequently than usual (response alternatives; no, yes, do not know). In accordance with the Rome IV criteria, nurses were classified as having IBS when endorsing abdominal pain at least one day a week, with duration of pain being over 6 months and when responding yes to at least two of the three criteria related to defecation (onset associated with improvement with defecation, or form of stool, or a change in frequency).

### Insomnia

The Bergen Insomnia Scale (BIS) was used to assess symptoms of insomnia [26]. BIS consists of 6 items, with response alternatives reflecting the number of days per week (zero to seven) where the respondent experiences a specific insomnia symptom. The questions refer to sleep onset (sleep latency exceeding 30 min), wake after sleep onset (more than 30 min), early morning awakening (more than 30 min), non-restorative sleep, daytime impairment, and dissatisfaction with sleep. Originally, the time frame in BIS was the past month [26]. However, in the present study this was modified to a time frame of the last three months based on the updated DSM-5/International Classification of Sleep Disorders-3 (ICSD-3) diagnostic criteria that changed in 2013 [27, 28]. According to the DSM-5/ICSD-3 criteria and based on the modified BIS questionnaire, insomnia disorder, was defined as scoring 3 days per week or more on at least one of the first three items, as well as 3 days per week or more on at least one of the latter two items. In addition, a total composite score (0 to 42) was calculated based on the scores of each item, where higher values indicate a greater degree of insomnia symptoms [26].

### Sleepiness

Sleepiness was measured with the Norwegian version [29] of the Epworth Sleepiness Scale (ESS) [30]. The ESS consists of 8 items measuring the subject’s general tendency to sleep or doze off in different situations. Each item is scored from 0 (no probability) to 3 (high probability), yielding a total score between 0 and 24, where higher score is associated with increased sleepiness. Excessive sleepiness was defined as scoring 11 or higher on the total score in accordance with the threshold described in the original publication of the instrument [30].

### Shift work disorder (SWD)

Shift work disorder was measured with 3 questions based on the criteria found in the ICSD-3 [10] and nurses were classified as having SWD when endorsing all three; (a) Do you have a work schedule that sometimes overlap with the time you usually sleep?, (b) if yes, does this cause insomnia and/or excessive sleepiness due to reduced amount of sleep?, (c) if yes, has this lasted for at least three months? The questions have previous been used in several studies [31–33].

### Statistics

IBM SPSS Statistics 28.0.1.0 for Windows was used for the statistical analyses.

The association between categorical variables of insomnia, excessive sleepiness, shift work disorder, different shift work categories and IBS was explored using Pearson chi-square tests. Both crude and adjusted (for sex and age due to their influence on sleep and IBS) binary logistic regression analyses were conducted with IBS (not IBS = 0 and IBS = 1) as dependent variable and work-related factors, insomnia, sleepiness and SWD as independent variables. Model parameters were reported in terms of overall chi-square values, degrees of freedom, p-value and Nagelkerke R-square values. The Hosmer and Lemeshow test was used for assessing the goodness-of-fit of the models. The assumption regarding lack of multicollinearity was investigated for all models. The linearity assumption was investigated by adding Box-Tidwell transformed continuous predictors to the relevant models.

IBS was set at the value 1 if responding yes to having abdominal pain at least one day a week, with duration of pain being over 6 months and when endorsing at least two of the three criteria related to defecation. SWD was set at 1 when answering yes on all three questions; (a) Do you have a work schedule that sometimes overlap with the time you usually sleep?, (b) if yes, does this cause insomnia and/or excessive sleepiness due to reduced amount of sleep?, (c) if yes, has this lasted for at least three months? Significance level was set to 0.05.

### Ethics

The study was approved by the Regional Committee for Medical and Health Research Ethics of Western Norway (REK-West, no 088.08). The study was carried out in accordance with relevant guidelines and regulations, and informed written consent was obtained from all participants included in the study.

## Results

Of the total sample, 90.4% were females. The mean age was 41.5 years ranging from 30 to 69. Table 1 presents the work schedule characteristics of the study sample. Just about one third of the nurses were day only workers and similar numbers were two-shift and three-shift workers. Mean time working as a nurse was 13.8 years (SD 4.1), ranging from 10 to 39 years.

**Table 1:** Work schedule, numbers of night work and quick returns worked the last year of the 1335 nurses included in the study

Work schedule (n=1308)	
Day only (n=377)	28.80%
Two-shift (n=405)	31.00%
Three-shift (n=383)	29.30%
Night only (n=87)	6.70%
Other schedules with night work (n=56)	4.30%
Number of night shifts last year, mean (SD) (n=1317)	19.7 (35.6)
Number of nights shifts worked the last year in groups	
0 (n=667)	50.60%
1-20 (n=279)	21.20%
>20 (n=371)	28.20%
Number of QRs last year, mean (SD) (n=1306)	27.6 (32.2)
Number of QRs worked the last year in groups	
0 (n=407)	31.20%
1-20 (n=334)	25.60%
>20 (n=565)	43.30%

SD=Standard deviation. QR=Quick returns

A total of 6.3% of the nurses reported IBS according to the Rome IV criteria. For the sleep parameters, 30.9% of the nurses reported insomnia as measured by the BIS and 25.6% reported excessive sleepiness measured by the ESS. The mean BIS total score was 11.9, and the mean ESS score was 7.9. A total of 33.6% of the nurses reported having SWD.

One of the models (including night work as a dichotomized variable) had poor Hosmer and Lemeshow goodness-of-fit (*p* <.05) and were consequently discarded, whereas all the other models had acceptable goodness-of-fit (*p* >.05). For all the adjusted logistic regression analyses the variance inflation factor was less than 2, hence no violation of the lack of multicollinearity assumption was detected. Further, none of the Box-Tidwell transformed continuous predictors turned out significant, indicating no violation of the linearity assumption.

IBS was not more prevalent among nurses having a work schedule including night shifts (day work/night work) (5.9% vs. 7.2%, *p*=.351), or among any of the four shift work schedules: day shift only (7.0%), two-shift (4.8%), night shift only (4.6%) or three-shift (8.1%), *p*=.259. The prevalence of IBS was not different depending on number of night shifts (no night work 6.0% compared to 1–20 night shifts 6.9%, and > 20 night shifts 6.4%, *p*=.865), or number of QRs (no QRs 6.8% compared to 1–20 QRs 5.2%, and > 20 QRs 6.7%, *p*=.623). However, IBS was more prevalent among nurses with insomnia (9.7%) than among those without (4.8%, *p*<.001), among those with excessive sleepiness (10.7% than those without (4.9%, *p*<.001), and among nurses with SWD (9.1%) compared to nurses without (5.0%, *p*=.004). Interpretation of the null findings relied on effect sizes and confidence intervals, which suggested that any associations with work schedule variables were small.

The results from the logistic regression analyses are presented in Table 2. Males had higher odds for reporting IBS compared to females (OR 2.16, 95% CI 0.78–5.99). When adjusting for sex and age, insomnia (aOR 2.14, 95% CI 1.36–3.67), excessive sleepiness (aOR 2.36, 95% CI 1.48–3.75) and shift work disorder (aOR 1.95, 95% CI 1.24–3.06) all remained significantly associated with IBS.

**Table 2:** Crude and adjusted logistic regression analyses with having IBS as the dependent variable among Norwegians nurses

	Having IBS OR (95% CI)^2^	Having IBS OR (95% CI)^3^	Model^4^
Sex (n=1298)			
Female^1^	1.00		
Male	2.16 (0.78-5.99)		
Age (n=1300)	1.00 (0.97-1.03)		
Work schedule with no night work^1^ (n=1280)	1		x^2^(3)=3.75, p=.295, R^2^=.008
Work schedule with night work	1.24 (0.79-1.94)	1.28 (0.81-2.01)	
Work schedule (n=1224)			
Day only^1^	1.00	1.00	
Two-shift	0.67 (0.37-1.24)	0.68 (0.37-1.26)	x^2^(5)=5.81, p=.325, R^2^=.005
Night only	0.64 (0.22-1.88)	0.68 (0.23-2.08)	
Three-shift	1.16 (0.67-2.01)	1.19 (0.69-2.06)	
Number of nights worked last year (n=1287)	1.00 (0.99-1.01)	1.00 (0.99-1.01)	x^2^(3)=2.76, p=.430, R^2^=.002
Number of nights (n=1287)			
0	1.00	1.00	x^2^(4)=3.05, p=.550, R^2^=.006
1-20	1.17 (0.66-2.06)	1.19 (0.67-2.10)	
>20	1.07 (0.63-1.83)	1.12 (0.65-1.90)	
Number of QRs worked last year (n=1276)	1.01 (1.00-1.01)	1.00 (0.99-1.01)	x^2^(3)=3.89, p=.274, R^2^=.008
Number of QRs (n=1276)			
0	1.00	1.00	x^2^(4)=3.71, p=.447, R^2^=.008
1-21	0.76 (0.41-1.42)	0.76 (0.41-1.43)	
>20	0.99 (0.59-1.67)	1.01 (0.60-1.69)	
Insomnia (n=1303)			x^2^(3)=13.18, p=.004, R^2^=.027
No insomnia^1^	1.00		
Insomnia	2.13 (1.36-3.34)	**2.14 (1.36-3.37)**	
Sum BIS (n=1289)	1.05 (1.03-1.08)	**1.05 (1.03-1.08**)	x^2^(3)=18.07, p=.004, R^2^=.037
Sleepiness (n=1264)			
ESS ≤10^1^	1.00		x^2^(3)=14.86, p=.002, R^2^=.031
ESS >10	2.33 (1.47-3.70)	**2.36 (1.48-3.75)**	
Sum ESS (n=1264)	1.13 (1.07-1.19)	**1.13 (1.07-1.20)**	x^2^(3)=20.76, p>.001, R^2^=.043
Shift Work Disorder (n=1297)			
No SWD^1^	1.00		x^2^(3)=10.94, p>.012, R^2^=.022
SWD	1.91 (1.22-2.30)	**1.95 (1.24-3.06)**	

^1^Comprised the reference/contrast group

^2^Separate crude logistic regression analyses for each independent variable

^3^Separate logistic regression analyses for each independent variable with adjustment for sex and age

^4^Nagelkerke pseudo R

QR=Quick returns, BIS=Bergen Insomnia Scale, ESS=Epworth Sleepiness Scale, SWD=Shift Work Disorder, IBS=Irritable Bowel Syndrome, CI=Confidence Interval, OR=Odds ratio

Significant findings are shown in **bold**

## Discussion

In this cross-sectional study, just above 6% of the total sample of nurses reported to have irritable bowel syndrome (IBS). There was no difference in the prevalence of IBS among the nurses working in different shift work schedules. About one third of the population had insomnia, about one quarter reported excessive sleepiness, and about one third reported shift work disorder (SWD). Furthermore, IBS was more prevalent among nurses with these sleep related problems.

IBS is considered as a common condition in the general population, with even higher prevalence among females and younger people [34]. Higher prevalence is also reported among shift workers compared to day workers [12, 19]. Among the nurses in the present study, the overall prevalence of IBS was 6.3%, thus lower than what could be expected in shed of prior research considering that nearly 90% of the study population were females, relatively young, and because more than 70% of the nurses were engaged in two- or three shift work schedules or night work. However, global prevalence rates of IBS vary greatly between countries, much related to differences in food, culture but also methodological differences across studies [35, 36]. One major source of variation stems from the diagnostic criteria used to assess IBS. For instance, one meta-analysis from 2020 investigating the global prevalence of IBS, found that the prevalence was substantially lower when applying the Rome IV criteria compared to the Rome III criteria, suggesting that the most recent criteria are more conservative [37]. In the meta-analysis the pooled IBS prevalence among the studies using the Rome III criteria was 9.2% compared to 3.8% among the studies that used the Rome IV criteria [37]. Also, another recent multinational study including 33 countries reported global prevalence rates between 3% and 5% in most countries when using the Rome IV criteria, which are more aligned with the prevalence reported in the present study [38]. A meta-analysis including 3360 medical staff reported an overall prevalence of IBS of 16%, and that the prevalence of nurses was higher compared to medical doctors [39]. However, most of the included studies were based on the rather lenient Rome III criteria [39]. Taken together, the observed overall prevalence of the present study of 6.3% in this nurse population is therefore higher than what has been reported globally in the general adult population using the same IBS criteria, but lower than what found in other shift work populations, likely reflecting both diagnostic stringency and methodological rigor. These differences between prevalences underline the impact of stricter symptom thresholds and revised definitions in Rome IV, which likely contribute to lower prevalence estimates.

Our findings did not lend support to the first hypothesis, that nurses with work schedules resulting in disrupted circadian rhythms would report higher prevalence of IBS compared to nurses with work schedules that do not disrupt circadian rhythms, suggesting alternative biological or psychosocial mechanisms may play a more prominent role in the symptom development. Nevertheless, the prevalence of IBS within the different work schedules in the study showed lower prevalences among the workers not rotating in the work schedule (night only 4.6%, day only 7.0%, and day/evening 4.8%) and the highest among the three shift workers (8.1%), yet this difference was not statistically significant. Our finding stands in contrast to the meta-analysis by Wang and colleagues concluding that shift work is an independent risk factor for IBS [22]. However, all studies included in that meta-analysis used the Rome II or Rome III criteria in contrast to the present study using the more stringent Rome IV criteria. Also, the meta-analysis of Liu et al. [39] reported higher prevalences among medical staff than reported in the present study, with highest numbers among nurses, suggesting that shift work, poor sleep quality and female gender were the influencing factors. Still, our results are in line with the study by Wells et al. on medical students doing overnight call shifts where no association with IBS was found [21].

In line with other studies among health care workers, the nurses in the present study reported high levels of insomnia, excessive sleepiness and shift work disorder [40]. About 31% of the nurses in the present study reported insomnia, more than 25% had excessive sleepiness and about 33% had SWD. IBS was more prevalent among nurses with insomnia, excessive sleepiness and among those with SWD. This supported our second hypothesis that nurses with sleep or sleepiness problems would report higher prevalence of IBS compared to nurses not having such problems. Nurses with IBS had higher odds of reporting insomnia, excessive sleepiness and SWD in the logistic regression analyses, also after adjusting for sex and age compared to nurses without IBS. Our findings are as such in line with several other studies of nurses where IBS is found to be significantly more common in nurses with poor sleep [17, 41]. However, the prevalence of IBS among the nurses with insomnia, excessive sleepiness and SWD in the present study was lower than for instance in the study among Chinese nurses showing that more than 35.2% of the nurses with poor sleep had IBS [41] compared to 9.3% of the nurses with poor sleep in the present study. This higher prevalence among the Chinese nurses may be due to the use of different measures of both sleep and IBS, as the study from Zhou and colleagues measured sleep using the Pittsburgh Sleep Quality Index [42], and used the less stringent Rome III criteria to assess IBS. Still, the mechanisms linking sleep disorders with IBS remain unclear, but gut-brain-microbiotic axis has been hypothesized to play an important role [17].

## Strengths and limitations

Some strengths of this study deserve mention. First, the study consists of a relatively large sample size comprising a homogenous group of shift workers. Also, the study includes different details about work-related factors such as work schedule, number of night shifts and quick returns, and standardized and widely used instruments for the sleep parameters assessing insomnia, sleepiness and SWD. We also used the latest and most stringent criteria for assessing IBS.

In terms of limitations, it should be noted that the initial response rate at baseline in this cohort was low, but still regarded as acceptable for such studies [43]. The nurses in the study had long experience working as nurses, with a mean of nearly 14 years. Thus, the results may not be representative for newly educated nurses, or nurses with little work experience. Other limitations include potential selection biases like the healthy worker effect. In addition to the fact that nurses who participate in research are often healthier and more resilient than the general population or their non-participating counterparts, participants in cohorts may have lower prevalences of chronic diseases such as IBS compared to their peers. Accordingly, we cannot rule out that nurses with severe symptoms of IBS or problems related to shift work have stopped working as nurses and thus are excluded from the present study. Still, this potential bias is not unique to the present study and may also be present in prior research reporting higher prevalence rates. Also, we cannot rule out that nurses with shift work related sleep or sleepiness problems, have changed to day work, as the mean experience as a nurse was long. Further, as the study was based on a large and homogeneous sample of nurses, with a large female preponderance, this puts limit on the generalizability of the findings to other occupations, nationalities and to males. The data were solely based on questionnaire data and self-reports, and the study did not include any clinical measures to verify neither the sleep and/or sleepiness problems nor the IBS symptoms that should be noted as important limitations. The fact that all substantially important measures were administered at the same time renders the findings susceptible to the common method bias, which may cause inflated relationships between study variables [44]. Also, information about working time was based on self-report rather than objective work records. Such reliance of self-report may introduce recall bias and reduce the accuracy of exposure assessment. Additionally, insomnia, excessive sleepiness, and SWD share overlapping symptoms. Multicollinearity diagnostics indicated however no statistical violation. Further, zero order correlations (phi-coefficients) between the three constructs were calculated (range 0.170 − 0.184) suggesting absence of problematic statistical overlap. Still, the results should be interpreted with awareness of potential conceptual overlap and shared variance. The instrument to assess SWD is not validated, though its limited psychometric validation may introduce measurement error and potential misclassification bias. It should also be noted as a limitation that the cross-sectional data in our study did not allow for causal inferences. Although the regression included several relevant variables, other variables such as dietary habits, stress, work demands, medication use, comorbid somatic/psychiatric conditions, BMI and other could potentially affect the relationship between shift work and IBS. In this regard it should be noted that the explained variance was low (R2=0.002-0.043), suggesting that other relevant independent variables were not measured/included in the current study. Stress is known to influence immune function and gut-brain axis signalling, potentially exacerbating intestinal inflammation and symptom severity. Workers with IBS report exposure to higher levels of occupational stressors and more work productivity impairments compared to workers without IBS. Regarding the association between characteristics of work and IBS, both shift work and job demands have, among others, been associated with IBS [45]. Including validated stress and emotional assessments could have strengthened the analysis by accounting for potential neuroimmune modulation and identified correlations between psychological stress and disease activity. Hence, one noteworthy limitation of the present study is the absence of stress measurements, which may have provided valuable insight into the role of psychosocial factors in IBS, particularly given the established influence of stress on gut-brain interactions and symptom worsening. Lastly, another limitation was that we did not perform any a priori power calculations.

## Conclusions

The prevalence of IBS in this population of nurses was quite low and did not show a clear association with the shift work schedule. However, IBS was more prevalent among nurses having sleep problems like insomnia, excessive sleepiness and shift work disorder. When adjusted for sex and age, insomnia, excessive sleepiness and shift work disorder were associated with two-fold higher odds of IBS among these Norwegian nurses. To ensure both nurse well-being and patient safety, the healthcare system should optimize the shift schedules by including recommendations based on knowledge about circadian rhythms, sleep health and psychosocial well-being, as well as individual differences/preferences.

## Data Availability

The datasets used and/or analysed during the current study are available from the corresponding author on reasonable request.

## Abbreviations

IBS: Irritable bowel syndrome
SUSSH: SUrvey of Shift work, Sleep and Health
BIS: Bergen Insomnia Scale
ESS: Epworth Sleepiness Scale
SWD: Shift Work Disorder
QRs: Quick Returns
GI: Gastrointestinal
NNO: Norwegian Nurses Organisation
ICSD: International Classification of Sleep Disorders
